# Application of a Phage Cocktail for Control of *Salmonella* in Foods and Reducing Biofilms

**DOI:** 10.3390/v11090841

**Published:** 2019-09-10

**Authors:** Md. Sharifull Islam, Yang Zhou, Lu Liang, Ishatur Nime, Kun Liu, Ting Yan, Xiaohong Wang, Jinquan Li

**Affiliations:** 1 Key Laboratory of Environment Correlative Dietology, College of Food Science and Technology, Huazhong Agricultural University, Wuhan 430070, China; 2 State Key Laboratory of Agricultural Microbiology, Huazhong Agricultural University, Wuhan 430070, China; 3 College of Fisheries, Huazhong Agricultural University, Wuhan 430070, China; 4 Division of Food Sciences, University of Nottingham, Sutton Bonington Campus, Sutton Bonington, Leicestershire LE12 5RD, UK; 5 Laboratory of Bacterial Pathogenesis and Immunology, The Rockefeller University, New York, NY 10065-6399, USA

**Keywords:** phage, cocktail, *Salmonella*, biological control, foods, biofilms

## Abstract

*Salmonella* contamination in foods and their formation of biofilms in food processing facility are the primary bacterial cause of a significant number of foodborne outbreaks and infections. Broad lytic phages are promising alternatives to conventional technologies for pathogen biocontrol in food matrices and reducing biofilms. In this study, 42 *Salmonella* phages were isolated from environmentally-sourced water samples. We characterized the host range and lytic capacity of phages LPSTLL, LPST94 and LPST153 against *Salmonella* spp., and all showed a wide host range and broad lytic activity. Electron microscopy analysis indicated that LPSTLL, LPST94, and LPST153 belonged to the family of *Siphoviridae*, *Ackermannviridae* and *Podoviridae*, respectively. We established a phage cocktail containing three phages (LPSTLL, LPST94 and LPST153) that had broad spectrum to lyse diverse *Salmonella* serovars. A significant decrease was observed in *Salmonella* with a viable count of 3 log_10_ CFU in milk and chicken breast at either 25 °C or 4 °C. It was found that treatment with phage cocktail was able to significantly reduced biofilm on a 96-well microplate (44–63%) and on a stainless steel surface (5.23 to 6.42 log_10_). These findings demonstrated that the phage cocktail described in this study can be potentially used as a biological control agent against *Salmonella* in food products and also has the effect to reduce *Salmonella* formed biofilms.

## 1. Introduction

*Salmonella* is gram-negative, rod-shaped bacterium that belongs to the family of *Enterobacteriaceae*. It is one of the most common food-borne pathogens, and has been considered as a significant public health threat and economic burden. It is a facultative intracellular human pathogen and the causative agent of non-typhoidal salmonellosis [1,2]. The symptoms of the disease are abdominal pain, vomiting, inflammatory diarrhea, nausea, fever, headache and the disease is frequently transmitted via contaminated foods, water and biofilms [3,4]. *Salmonella* outbreaks by consuming contaminated food products were previously reported, and chicken, pork, beef and dairy products were common vehicles [5,6]. It is estimated that every year *Salmonella* spp. cause 93.8 million illnesses and 155,000 deaths worldwide [7]. According to USDA-ERS estimation, economically, US $2.5 million was lost due to 1.4 million cases of salmonellosis in 2007 [8]. In the United States alone, *Salmonella* is annually responsible for 11% of illnesses, 35% of total hospitalizations and 28% of deaths associated with food-borne diseases [9,10]. In addition, over 265 people were sickened owing to consumption of chicken salad that was contaminated by *Salmonella* in eight states in the USA in 2018. Among them, 94 were hospitalized and one person died [11]. In China, non-typhoidal *Salmonella* spp. are extremely important. From 2008 to 2012, approximately 70 outbreaks were reported, which led to 4151 hospitalized cases and four deaths with many provinces affected [12,13,14].

*Salmonella* spp. are frequently described as environmental persisters [15,16] and can form surface-associated complex communities known as biofilms in both food matrices and industrial settings [17,18,19,20]. *Salmonella* biofilm was reported as bacterial reservoir in a food processing facility, and lead to several food-borne disease outbreaks [21]. For example, *Salmonella* outbreaks that caused 2138 cases of illness was related to the consumption of *Salmonella* biofilm-contaminated chicken [22].

Conventional control measures such as chemical disinfectant, biocides, and heat treatment are frequently used against various *Salmonella* serovars, in food products and to reduce biofilms [23,24,25]. However, they all possess certain disadvantages. Chemical disinfectants such as ascorbic acid, calcium carbonate and diacetyl-tartaric esters of fatty acids have an adverse impact on the taste, aroma, and texture, which are very desirable traits of foods [26]. Chemical preservatives such as sodium benzoate and benzoic acid could also lead to number of side effects such as asthma, allergic contact dermatitis, hives, convulsions and intestinal hemorrhage diarrhea [27,28]. Moreover, general disinfectants (sodium hypochlorite, sodium hydroxide, and benzalkonium chloride) failed to reduce *Salmonella enterica* biofilms [21] due to high bacterial resistance [29]. The heat treatment can destroy vitamins thus reducing the nutritional value of foods [30,31] and also produce advanced glycation end-products (AGEs) attributed to health-threatening complications [32].

As most conventional methods showed limited effect on *Salmonella* control, antibiotics once were considered as an effective method to reduce the *Salmonella* burden in both farms and industries. However, later this was proved as leading to another issue, the prevalence of antimicrobial resistant bacteria. Antibiotics have been banned from Sweden in 1986, the Danish Pig Production Committee (NCPP) in 1995 and the European Union (EU) in 1999 [33]. As a novel strategy, phages were described as a promising approach to control and preserve pathogens in food products and to reduce biofilms [34,35,36,37]. Human and animal bodies are reservoirs for great amounts of phages. As reported, phages can easily be detected from healthy humans, animals, and foods [38,39,40,41]. Phages do not cause harm to humans and animals and there have been no reports so far describing any phage infection in the human body [38,39]. Thus, phage application seems to be a safe prerequisite to biologically control pathogens in food processing line [42].

Bacteriophages are frequently used for inactivation and control of food-borne pathogens, such as *Salmonella*, *Escherichia coli* O157:H7, *Listeria* and *Campylobacter* in different foods [43,44,45,46,47,48,49,50,51,52,53,54]. Several physical, chemical, and biological approaches such as cold oxygen plasma, ultraviolet irradiation, ultrasound, natural substances, quorum sensing inhibition, antimicrobial coating, and bacteriophages have been proposed as tools to control biofilms in the food industry. However, most of these strategies are either limited with efficacy, not cost-effective, or not practical for implementation in food processing facilities [55]. Among these advanced approaches, bacteriophages are considered as potential candidates to reduce or eliminate biofilms [37]. Few studies have been conducted on the effectiveness of phage against biofilms formed by *Salmonella* spp. on food or food processing surfaces. This study aims to evaluate the efficacy of phage cocktail as a zoonotic *Salmonella* control approach in diverse foods and biofilms.

## 2. Materials and Methods

### 2.1. Bacterial Strains and Culture Conditions

A selection of 55 different bacterial strains, including 41 *Salmonella* strains that encompassed in 11 distinct serovars, and a cohort of 14 non-*Salmonella* strains (including *E. coli A. hydrophila, S. aureus* and *Listeria*), were used in this study to screen phage by spot test (Table 1). These strains were stored with 20% (*v*/*v*) glycerol at −80 °C. All bacterial strains were streaked on tryptic soy agar (TSA; Difco^TM^, BD, Franklin Lakes, NJ, USA) before the experiment, and obtained single colonies were recovered by culturing in tryptic soy broth (TSB, Difco^TM^, BD, USA) overnight at 37 °C to ensure the purity of the bacterial stock. *Salmonella enterica* was used for isolation, propagation, and purification of phages. The phages were isolated and enriched using 2-YT broth medium (1.6 g of peptone, 1.0 g of yeast extract, and 0.5 g NaCl, in 100 mL of distilled water; pH 7.4). To determine the phage titer, a double-layer agar plates method was applied, with an overlay layer containing 0.7% agar and a bottom layer with 1.5% agar [54].

### 2.2. Enrichment, Isolation, Purification, and Preparation of Phages

A total of 42 phages were isolated from environmentally sourced water samples collected in Wuhan, China in accordance with previously described method [56]. For enrichment, isolation and purification of phages, modified methods from previously published articles were used [57,58,59]. In brief, 10 mL of a 0.22-μm-filtered sample was mixed with 40 mL 2-YT broth medium and 10 mL exponential growth phase *Salmonella* cultures at a ratio of 1:4:1 (*v*/*v*/*v*). After 24 h of incubation at 37 °C with gentle agitation, the cultured was centrifuged (8000× *g*/15 min) and filtered again using 0.22-μm filters (Millipore, Dublin, Ireland). Then phage activity in the supernatant was detected with spot assay [54,60]. The double layer agar method was used to determine the titer of the phage stock. Dilutions of the phage stock (100 μL each) were made in sterile SM buffer (10 mM NaCl, 10 mM MgSO_4_, 50 mM Tris:HCl, pH 7.5), mixed with a suspension of exponential phase *Salmonella* (about 10^9^ CFU/mL, 100 μL) and added to 4 mL of molten (45 °C ≤ temperature ≤ 50 °C) TSB agar (0.7%). The mixtures were then poured onto the surface of TSA agar plates and were allowed to set at room temperature for 5 min. Thereafter, the plates were incubated at 37 °C for 24 h, and resulting plaques were quantified. To purify the phages, picking individual plaque by using a pipette or a wire loop, and then suspended in TSB with exponential phase *Salmonella* at 37 °C for 24 h. Then centrifuged (8000× *g*/15 min) and filtered again using 0.22-μm filters used as a single phage culture. The purification process was repeated at least three times, and then confirmed pure individual phage stock. Purified phages were stored at 4 °C and used for different experiments during the whole study.

The phage cocktail was prepared by mixing three phages with a ratio of 1:1:1, each phage at a titer of 9 log_10_ PFU/mL. The phage cocktail was later diluted in sterile SM buffer to reach the target concentration for treatment of *Salmonella* in foods and biofilms.

### 2.3. Screening of Phages Based on Spot Test and Lytic Capacity

#### 2.3.1. Spot Test

Spot testing was applied to measure the ability of phages to infect different serovars of bacteria with a modified method from previous publication [54]. 100 μL of test bacterial culture that are in exponential phase was transferred to 4 mL of molten (45 °C ≤ temperature ≤ 50 °C) TSB agar (0.7% *w*/*v*). The mixture was then poured onto surface of TSA agar plates and allowed to dry for 5 min. When the overlay agar was set, 5 μL of each phage solution was spotted onto bacterial lawns and allowed to dry. The plates were then incubated at 37 °C for 20 to 24 h. After incubation, any bacterial lawn with formation of clear spots/plaques were considered as phage sensitive.

#### 2.3.2. Lytic Activity

Phage lytic activity was analyzed in the 96-well microtiter plate by measuring the optical density (OD_600nm_) every hour with various applied multiplicity of infection (MOI; ratio of phage titers to bacterial counts measured) to determine the efficiency of phage virulence according to previously described method [61]. In brief, the test group with 100 μL of fresh cultured *Salmonella* (7 log_10_ CFU/mL) was added to 100 μL of individually diluted phage lysate (6 log_10_–9 log_10_ PFU/mL) in wells of 96 well-mirotitre plate. The control group was set up with same volume of fresh overnight cultures of *Salmonella* (7 log_10_ CFU/mL) mixed with plain TSB medium instead of phage. Samples were incubated at 37 °C on an orbital shaker at 160 rpm. Optical density (OD_600nm_) of the mixture was measured with a microplate reader (Infinite M200 Pro, Tecan, 140 Switzerland) at 37 °C, with an interval of 1 h.

### 2.4. Determination of Host Range by Efficiency of Plating (EOP)

The three phages that revealed widest bactericide host range in spot assays (LPSTLL, LPST94 and LPST153) were analyzed with efficiency of plating (EOP) either individually or assayed as a cocktail with ratio of 1:1:1. To evaluate the host range, EOP was performed as modified methods described in previous reports [61,62]. Each phage was serially diluted and tested in triplicates on sensitive bacterial host. Test bacterial strains were grown overnight at 37 °C. After incubation, 100 μL of bacterial culture was applied in double layer plate assays together with 100 μL of diluted phage lysate. Dilution factors between 10^6^–10^9^ were applied in this study. The plates were incubated overnight at 37 °C and the number of plaque forming units (PFU) was counted. The EOP was calculated (average PFU on test bacteria / average PFU on host bacteria). The average EOP value was classified as EOP 0.5 to 1.0, high efficiency; EOP 0.2 to <0.5, moderate efficiency; 0.001 to <0.2, low efficiency; and <0.001, inefficient [61,62].

### 2.5. Transmission Electron Microscopy (TEM)

Ten microliter lysate with a high titer (>10 log_10_ PFU/mL) of purified phage was fixed onto a copper grid and negatively stained with 0.5% phosphotungstic acid (PTA) [56,62,63]. Thereafter, negatively stained copper grids were examined, and the images of phage were captured using a Philips CM12 transmission electron microscope (Hitachi H-7000FA, Tokyo, Japan), at Wuhan Institute of Virology (China Academy of Sciences, Wuhan, China) and analyzed via Digital Micrograph Demo 3.9.1 software (Gatan, Pleasanton, CA, USA).

### 2.6. Phage Stability in Foods

Stability of phage cocktail in milk and on chicken breast experiments were conducted. Briefly, phage lysates were firstly added in milk to reach a final titer of 6 log_10_ and 7 log_10_ PFU/mL. Phages were also applied on chicken breast to a final titer of 6 log_10_ and 7 log_10_ PFU/cm2 by pipette transferring the lysate onto the surface of the chicken breast, followed by spreading the lysate with a sterile spreader. Both inoculated samples (milk and chicken breast) were incubated at 25 °C or 4 °C for 48 h. At each time-point (0, 1, 3, 6, 12, 24 and 48 h), aliquoted milk or pre-cut chicken breast were taken to enumerate phage titer using a double-layer agar method.

### 2.7. Biological Control of Salmonella in Foods Using Phage Cocktail

#### 2.7.1. Food Samples

Pasteurized milk was purchased from a local supermarket. The chicken breast was also obtained from a local supermarket then sliced aseptically in the laboratory. The chicken breast was cut into pieces (1 cm × 1 cm square) using a sterile scalpel blade on the sterile station board. Food samples were general screened with TSA for the presence of any microorganisms and only those ones that are free from background microorganisms were used in this study.

#### 2.7.2. Adding Salmonella and Phage Cocktail for Treatment

*Salmonella* biocontrol experiments using phage cocktail were conducted at 4 °C (refrigerator temperature) and 25 °C (room temperature) [64]. Study groups were temperature acclimated for 20 min; thereafter *S.* Typhimurium ATCC 14028 or a mixture of both *S.* Typhimurium ATCC 14028 and *S.* Enteritidis ATCC 13076 was added to milk to a final viable count of 3 log_10_ CFU/mL. The 1 cm × 1 cm square chicken breast sections were placed in the center of the sterile Petri-dishes, 3 log_10_ CFU/cm^2^ (final viable count) *Salmonella* suspension was added and spread over the sample surfaces. The chicken breast samples were dried for 30 to 40 min.

For phage cocktail treatment, phage cocktail was added to MOI of 1000 (add 10 μL of 8 log_10_ PFU/mL phage to reach a final titer of 6 log_10_ PFU/mL) or 10000 (add 10 μL of 9 log_10_ PFU/mL phage to reach a final titer of 7 log_10_ PFU/mL) in milk. phage cocktail was added with an MOI of 1000 (spot 10 μL of 8 log_10_ PFU/mL phage to reach a final titer of 6 log_10_ PFU/cm2) or MOI of 10000 (spot 10 μL of 9 log_10_ PFU/mL phage for a final titer of 7 log_10_ PFU/cm2) by pipette transferring the lysate followed by spreading the lysate with a sterile spreader on surface of the chicken breast samples. Then incubated at either 4 °C or 25 °C.

#### 2.7.3. Recovered Bacterial Load from Treated Foods

For the recovered bacterial load from milk, an aliquot of the samples was taken out after 0, 1, 3, 6, 12, 24 and 48 h of incubation at the corresponding temperature, and to avoid plating the bacteriophage, samples were centrifuged at 3000× *g* for 10 min [65] for bacterial precipitation and the supernatant containing phage was the discarded. Any changes in respective of the bacterial viable count in both control and experimental group were determined by adding 1 mL of PBS, followed by vigorous vortex, serial dilution and plating at each time point.

For the recovered bacterial load from chicken, pre-cut 1 cm^2^ chicken breast sample was taken out after 0, 1, 3, 6, 12, 24 and 48 h of incubation then sample was transferred to 2 mL Eppendorf tube and 1 mL PBS buffer was added to the sample in a sterile environment. The chicken sample was homogenized with sterile bars and vortexed [50]. To avoid plating the bacteriophage, 1 mL homogenized sample was centrifuged at 3000× *g* for 10 min [65] for bacterial precipitation and the supernatant containing phage was then discarded. Any changes in respective of the bacterial viable count in both control and experimental group were determined by adding 1 mL of PBS, followed by vigorous vortex, serial dilution and plating at each time point.

### 2.8. Effect of Phage Cocktail Against Biofilm of Salmonella in 96-Well Microplate and on Stainless Steel Surface

#### 2.8.1. Biofilm Assay in 96-Well Microplate

The colorimetric method was applied to quantitatively determine the effectiveness of the phage cocktail treatment on reducing biofilm of *Salmonella* according to the previously described method [66] with some adaptation. Overnight culture of *Salmonella* was prepared. In each well of the 96-well microplate, single *Salmonella* ATCC 14028 or a mixture of *Salmonella enterica* (ATCC 13076 and ATCC 14028) were inoculated into LB without NaCl, to reach a final viable count of 4 log_10_ CFU/mL. The microtiter plate was incubated at 30 °C (favorable temperature for biofilm formation) [67] for 72 h under static condition to allow bacteria to attach on well and further form biofilms. The medium was renewed every 24 h, followed by the phage cocktail treatment at a final titer of 7 log_10_ and 8 log_10_ PFU/mL. Phosphate-buffered saline (PBS) was used as control instead of phage. Samples were further incubated at 30 °C for 24 h. After phage cocktail treatment, each well was rinsed with PBS for 5 times and allow to air-dry. After rinse with PBS, 98% methanol was added, and left for 10 min. The methanol was then removed, and plates were air dried again. Samples were then stained with 1% crystal violet solution for 45 min followed by elution with 33% acetic acid. The OD of eluted sample was measured by a spectrometer at a wavelength of 600 nm. The biofilm reduction percentages were calculated according to the following formula [(C − B) − (T − B)]/[(C − B)] × 100 where C = average OD_600nm_ of the control group, B = average OD_600nm_ of blank wells containing test medium and T = average OD_600nm_ of phage-treated wells.

#### 2.8.2. Biofilm Assay on Stainless Steel Surface

The phage cocktail was tested for their ability to reduce biofilm cells from stainless steel (SS) coupons (1 cm × 1 cm square) according to published method [68] with some modification. The overnight bacterial culture was diluted 1:50 and inoculated into 10 mL LB without NaCl in 50 mL Falcon tubes; SS coupons were completely submerged in a Falcon tube containing 10 mL diluted bacteria to enable biofilm formation. The tubes were incubated without shaking at 30 °C for 72 h to enable development of biofilms on these coupons. Following coupons were transferred from the tube and washed five times with PBS to remove planktonic cells. The coupons were submerged in a tube containing 5 mL LB without NaCl and 5 mL phages solution with a final concentration of (7 log_10_ and 8 log_10_ CFU/mL) and incubated at 30 °C for 24 h. PBS used as a control instead of phage cocktail. Following incubation, SS coupons were rinsed with PBS 5 times and transferred to a sterile Petri dish that contained 1 mL of PBS, scrubbed, transferred to a test tube, and vortexed for 2 min to disperse the biofilm. The solution was centrifuged for 2 min at 12,000× *g* to separate bacteria from unabsorbed phage. Cells were diluted in PBS for counting. *Salmonella* was quantified by direct plating (viable count CFU/cm^2^). The logarithmic reduction of biofilm cells was calculated according to the following formula [log (untreated viable cell density)−log (treated viable cell density)].

### 2.9. Statistical Analysis

Food model assays and biofilm assays were done in triplicates and two samples per treatment were tested in each replicate. Results were reported as mean values of the three replicates with error bars suggesting the standard deviation of the mean. The bacterial and phage data were transformed to log_10_ units. The efficacy of phage cocktail in reducing the number of viable *Salmonella* in all foods and biofilms examined was evaluated by comparing the data obtained with the PBS-treated control samples to the phage cocktail-treated samples. Statistical analyses were performed by two-way analysis of variance (ANOVA) followed by Bonferroni’s test with 95% confidence interval using Prism 5.03 for Windows (GraphPad software, San Diego, CA, USA). Statistical significance was considered at significance level of *p* < 0.05.

## 3. Results

### 3.1. Isolation and Screening of Phages

A total of 42 different phages were isolated from environmentally sourced water samples using *S. enterica* as host. All isolated phages showed distinct difference in plaque size and turbidity from each other. All 42 isolated phages were able to lyse their host throughout the purification process. When these phages were screened by spot testing, 17% of isolated phages (7 out of 42) formed clear plaques and were capable to lyse at least two serovars (Table 2), whereas the rest of them were highly specific in infecting only their host. Spot test results showed that phages LPSTLL, LPST94, and LPST153 had broader host range compared to other phages (LPST81, LPST89, LPST109, and LPST115) isolated in this study (Table 2). Phages LPSTLL and LPST94 lysed all 41 (100%) tested strains that belong to 11 *Salmonella* serovars including drug-resistant *Salmonella*. Phage LPST153 lysed 50-100% strains of 9 *Salmonella* serovars (except 2 serovars; Newport and Kentucky). However, none of the phages isolated in this study were capable to lyse *E. coli* or other tested non-*Salmonella* bacteria. These results indicated that LPSTLL, LPST94 and LPST153 are *Salmonella*-specific and they all showed a broad lytic spectrum.

**Figure 1:**
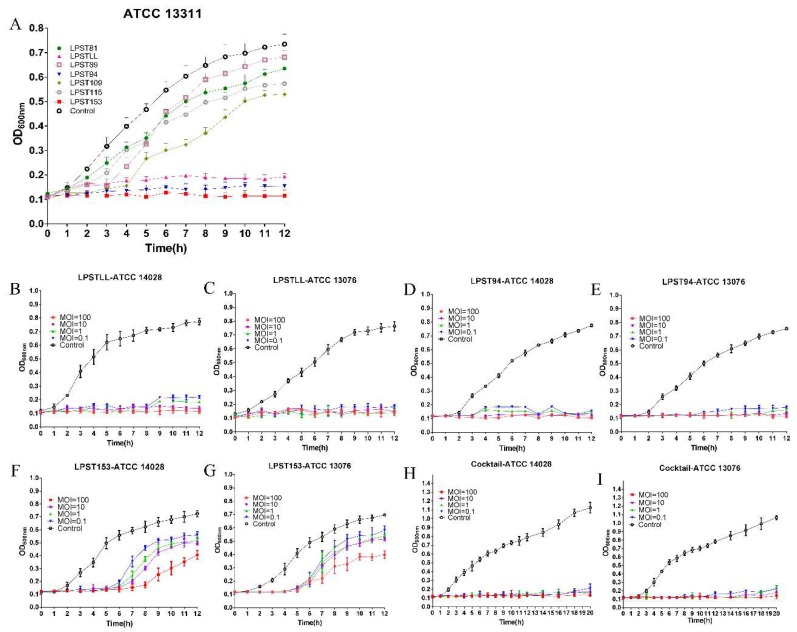
(**A**) Comparison of the lytic ability of selected phages using *S.* *enterica* serovar Typhimurium (ATCC 13311) as a host at MOI of 1 in TSB broth; Lytic ability of phage LPSTLL to lyse *S.* *enterica* serovar Typhimurium and *S.* *enterica* serovar Enteritidis in TSB medium at different MOIs of 100, 10, 1 and 0.1 at 37 °C in vitro: (**B**) *S.* *enterica* serovar Typhimurium ATCC 14028, (**C**) *S.* *enterica* serovar Enteritidis ATCC 13076; Lytic ability of phage LPST94 to lyse *S.* *enterica* serovar Typhimurium and *S.* *enterica* serovar Enteritidis in TSB medium at different MOIs of 100, 10, 1 and 0.1 at 37 °C in vitro: (**D**) *S.* *enterica* serovar Typhimurium ATCC 14028, (**E**) *S.* *enterica* serovar Enteritidis ATCC 13076; Lytic ability of phage LPST153 to lyse *S.* *enterica* serovar Typhimurium and *S.* *enterica* serovar Enteritidis in TSB medium at different MOIs of 100, 10, 1 and 0.1 at 37 °C in vitro: (**F**) *S.* *enterica* serovar Typhimurium ATCC 14028, (**G**) *S.* *enterica* serovar Enteritidis ATCC 13076; and Lytic ability of phage cocktail to lyse *S.* *enterica* serovar Typhimurium and *S.* *enterica* serovar Enteritidis in TSB medium at different MOIs of 100, 10, 1 and 0.1 at 37 °C in vitro: (**H**) *S.* *enterica* serovar Typhimurium ATCC 14028, (I) *S.* *enterica* serovar Enteritidis ATCC 13076. Values represent mean with standard deviation of three replicates of each time point.

In the process of selecting the most effective phages, further screening was carried out by lytic activity test. Lytic activity assay was conducted for 7 phages that could lyse more the 2 strains of *Salmonella* (Figure 1A). Inhibited growth of host bacteria (*S.* Typhimurium ATCC 13311) in 2 h was observed for all 7 tested phages. After 2 h, phages LPSTLL, LPST94 and LPST153 constantly inhibited the growth of host in 12 h whereas other phages (LPST81, LPST89, LPST109, and LPST115) lost the ability to reduce bacterial cell numbers, which resulted in commence of bacterial growth (Figure 1A). Results of lytic activity indicated that LPSTLL, LPST94 and LPST153 had strong lytic capacity. Those phages were selected for further conforming lytic activity and make the cocktail using this three phages. The lytic activity profile of LPSTLL, LPST94, LPST153 and phage cocktail against *Salmonella enterica* serovar Typhimurium (ATCC 14028) and *Salmonella enterica* serovar Enteritidis (ATCC 13076) were also generated at MOI of 0.1, 1, 10 and 100 to confirm the findings. Phages LPSTLL and LPST94 could constantly inhibit the growth of both *S.* Typhimurium and S. Enteritidis in 12 h and LPST153 could in 5 h (Figure 1B–G). Phage cocktail could constantly inhibit the growth of both *S.* Typhimurium and *S.* Enteritidis with low counts at MOI ratios of 0.1, 1, 10 and 100 in 20 h (Figure 1H,I). Phage cocktail extended bacterial inhibition and exhibited strong lytic ability; therefore it could be a potential candidate for the control of *Salmonella*.

### 3.2. Host Range of Phages by Efficiency of Plating (EOP)

Phages LPSTLL, LPST94, LPST153 and their phage cocktail mix were analyzed by EOP to confirm their host range (Table 3). Phage cocktail revealed the broadest spectrum of lytic activity compared with single phages LPSTLL, LPST94 and LPST153 isolated in our study. Phage cocktail had a moderate to high efficiency (0.2 to 1.0) to infect all (*N* = 41) of *Salmonella* strains including drug-resistant *Salmonella*. For the single phage efficiency, LPST94 showed the broadest spectrum of lytic activity. This phage had a high efficiency (0.5 to 1.0) to infect the majority of *S*. Typhimurium strains but the EOP values were moderate (0.2 to 0.5) for some *S.* Enteritidis and drug-resistant *Salmonella* strains. The LPST94 phage could also lyse all drug-resistance *Salmonella* strains (*N* = 19) in our collection, the EOP values ranged from 0.001 to 0.2. LPSTLL lysed all 7 strains of *S.* Typhimurium with EOP values (0.5 to 1.0) and LPST153 could lyse *S*. Typhimurium (*N* = 7), the EOP values (0.1 to 1.0). These two phages also could lyse all *S.* Enteritidis (*N* = 5), and EOP values were between 0.001 to <0.2. The EOP values were 0.001 to <0.2 or negative for other serovars. As previous reported, *S.* Enteritidis and *S.* Typhimurium are the most common serovars that could cause salmonellosis by contaminated foods [69]. These results suggested that the phage cocktail has a wide host range of lytic activity (Table 3).

### 3.3. Morphology of Phages and Stability of Phage Cocktail in Foods

**Figure 2:**
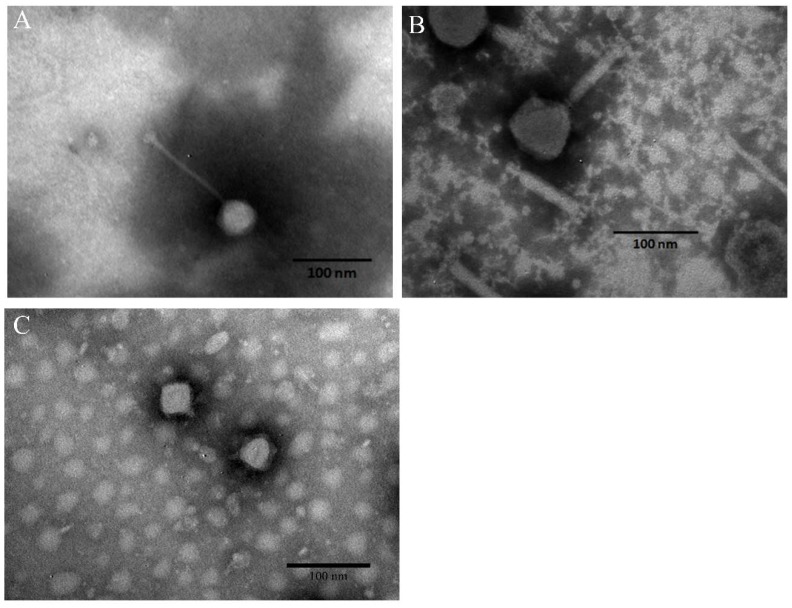
Morphological characteristics of phages. (**A**) TEM image of phage LPSTLL; (**B**) TEM image of phage LPST94 and (**C**) TEM image of phage LPST153. Bar 100 nm.

All three selected phages that were as identified tailed phages by transmission electron microscopic (TEM) and appeared to fall into the order of *Caudovirales* (Figure 2). Transmission electron microscopic examination of phage LPSTLL showed that it has isometric head with 55.27 ± 5.13 nm (*n* = 3) diameter, and a long non-contractile tail 126.8 ± 4.72 nm (*n* = 3) long (Figure 2A). These characteristics suggested that LPSTLL belonged to *Siphoviridae* family. Phage LPST94 had an icosahedral head, and a long, rigid and relatively thick contractile tail terminated in a baseplate with spikes as examined by TEM. The head of phage was 67.53 ± 2.20 nm in diameter (*n* = 3) and tail was 116.45 ± 4.05 nm long (*n* = 3) (Figure 2B). The morphology of the phage virion suggested that LPST94 belonged to the *Ackermannviridae* family. Phage LPST153 was found with short-tailed. The head diameter and tail lengths were 51.54 ± 4.70 nm and 7.33 ± 2.45 nm (*n* = 3), respectively (Figure 2C). TEM picture suggested that this phage belonged to the *Podoviridae* family. From the above results, it is suggested that the three selected phages belonged to three different families, *Siphoviridae, Ackermannviridae,* and *Podoviridae.*

**Figure 3:**
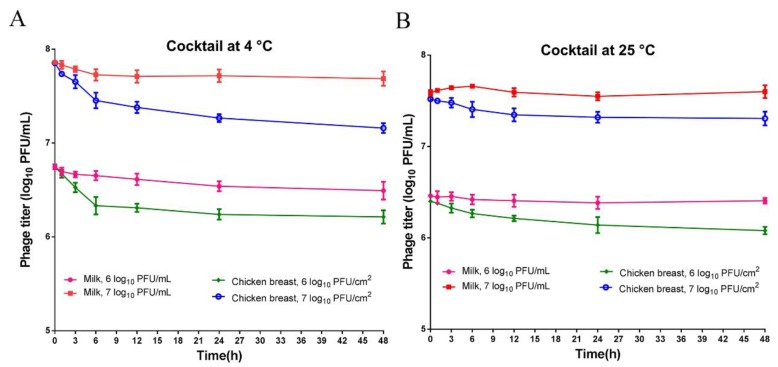
Stability of phage cocktail in foods (milk and chicken breast). Phage cocktail titer of 6 log_10_ PFU/mL and 7 log_10_ PFU/mL, incubated for 48 h at 4 °C (**A**) and 25 °C (**B**). Values represent mean with standard deviation of three replicates of each time point.

When phage cocktail was added to milk, and chicken breast, the titers remained stable with no inactivation observed at both 4 °C (Figure 3A) and 25 °C (Figure 3B). The results revealed that phage cocktail remained stable under the test conditions and could be a promising candidate to control *Salmonella* in foods.

### 3.4. Application of Phage Cocktail in Controlling Food-borne S. Typhimurium and S. Enteritidis

Phage cocktail composed of 1:1:1 mixture of phage LPSTLL, LPST94 and LPST153 was evaluated for biological control of experimentally contaminated milk and chicken breast. Samples were inoculated with either *S.* Typhimurium (ATCC 14028) at a final concentration 3 log_10_ CFU/mL alone, or a mixture of *Salmonella* (*S.* Typhimurium ATCC 14028 and *S.* Enteritidis ATCC 13076) culture at same concentration 3 log_10_ CFU/mL at 4 °C and 25 °C.

**Figure 4:**
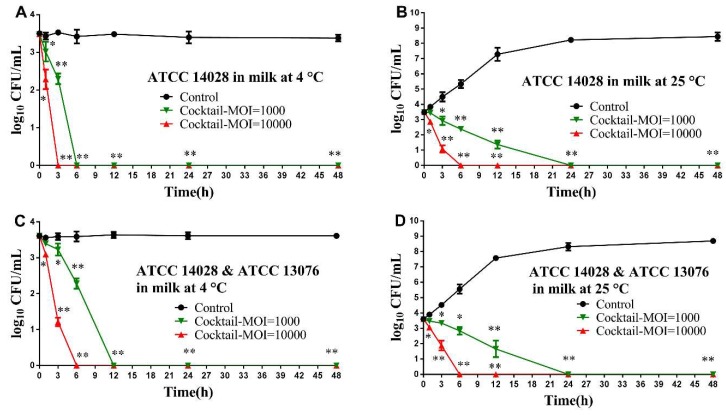
Effectiveness of phage cocktail in reducing the *S.* Typhimurium ATCC 14028 and *S.* Enteritidis ATCC 13076 in milk. (**A**) Effect of phage cocktail on growth of *S.* Typhimurium ATCC 14028 in milk at 4 °C; (**B**) Effect of phage cocktail on growth of *S.* Typhimurium ATCC 14028 in milk at 25 °C; (**C**) Effect of phage cocktail on growth of *Salmonella* mixture (*S.* Typhimurium ATCC 14028 and *S.* Enteritidis ATCC 13076) in milk at 4 °C and (**D**) Effect of phage cocktail on growth of *Salmonella* mixture (*S.* Typhimurium ATCC 14028 and *S.* Enteritidis ATCC 13076) in milk at 25 °C. Values represent mean with standard deviation of three determinations. ** Significant at *p* < 0.01; * Significant at *p* < 0.05.

In milk assay, the effectiveness of phage cocktail to reduce *Salmonella* was remarkable; the viable count of the *S.* Typhimurium in milk was reduced below detectable limit (<1 CFU/100 μL) after 3 h and 6 h at 4 °C using an MOI of 10000 and 1000, respectively (Figure 4A). While at an MOI of 10000 and 1000 for single *Salmonella* or a mixture of *Salmonella*, the viable counts declined completely after 6 h and 24 h, respectively, at 25 °C (Figure 4B & 4D). For treatment against the mixture of *Salmonella* (*S.* Typhimurium ATCC 14028 and *S.* Enteritidis ATCC 13076), there was almost a complete elimination of *Salmonella* in milk after 6 h and 12 h at 4 °C with an MOI of 10000 and 1000, respectively (Figure 4C).

**Figure 5:**
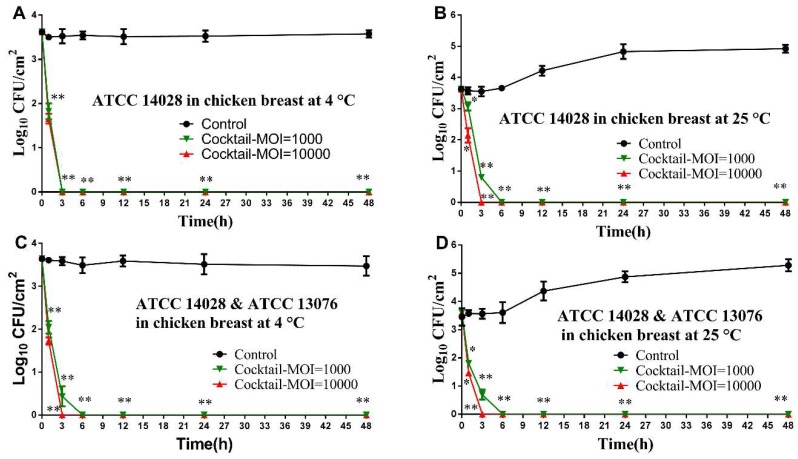
Effectiveness of phage cocktail in reducing the *S.* Typhimurium ATCC 14028 and *S.* Enteritidis ATCC 13076 in chicken breast. (**A**) Effect of phage cocktail on growth of *S.* Typhimurium ATCC 14028 in chicken breast at 4 °C; (**B**) Effect of phage cocktail on growth of *S.* Typhimurium ATCC 14028 in chicken breast at 25 °C; (**C**) Effect of phage cocktail on growth of *Salmonella* mixture (*S.* Typhimurium ATCC 14028 and *S.* Enteritidis ATCC 13076) in chicken breast at 4 °C; and Effect of phage cocktail on growth of *Salmonella* mixture (*S.* Typhimurium ATCC 14028 and (**D**) *S.* Enteritidis ATCC 13076) in chicken breast at 25 °C. Values represent mean with standard deviation of three determinations. ** Significant at *p* < 0.01; * Significant at *p* < 0.05.

The ability of phage cocktail to reduce the level of artificially contaminated *Salmonella* on chicken breast is also demonstrated, there are no viable count could be recovered by direct plating after 3 h incubation using both MOIs of 10000 and 1000 at 4 °C (Figure 5A). Similarly, at 25 °C, the *Salmonella* counts were eliminated completely after 3 h and 6 h upon application of phage cocktail at an MOI of 10000 and 1000, respectively (Figure 5B). Like the observations of *S.* Typhimurium ATCC 14028, at both 4 °C and 25 °C, administration of phage cocktail with an MOI of 10000 and 1000 revealed a similar trend of reduction of *Salmonella* mixture (Figure 5C,D).

### 3.5. Effect of Phage Cocktail Against biofilm of Salmonella

**Figure 6:**
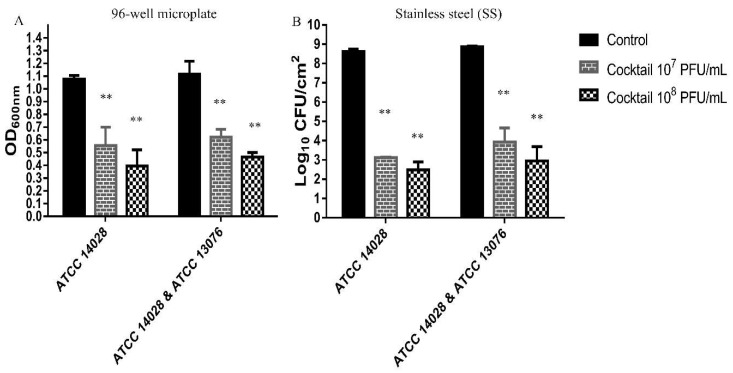
Effect of phage cocktail on biofilm. (**A**) Effect of phage cocktail on 72-h-old biofilm in 96-well microplate and (**B**) on stainless steel surface at 30 °C after 24-h post-infection. Values represent mean with standard deviation of five determinations. ** Significant at *p* < 0.01.

The efficacy of phage cocktail composed of 1:1:1 mixture of the phages LPSTLL, LPST94 and LPST153 against biofilm of *S.* Typhimurium (ATCC 14028) alone or a mixture of *Salmonella* (*S.* Typhimurium ATCC 14028 and *S.* Enteritidis ATCC 13076) in 96-well microplate or on stainless steel (SS) surface were evaluated at 30 °C (favorable temperature for biofilm formation) [67]. Figure 6 shows the reduction of biofilm after phage cocktail treatment with titers of 7 log_10_ PFU/mL and 8 log_10_ PFU/mL for 24 h. Treatment of *S.* Typhimurium (ATCC 14028) alone or a mixture of Salmonella (*S.* Typhimurium ATCC 14028 and *S.* Enteritidis ATCC 13076) with phage cocktail reduced the biofilm in 96-well microplate or on stainless steel (SS) surface significantly (*p* < 0.01). In 96-well microplate, the single *S.* Typhimurium (ATCC 14028) biofilm removal activity of 48.3% and 63.25% were respectively observed when phage cocktail was applied to a final titre of 7 log_10_ and 8 log_10_ PFU/mL (Figure 6A). For the mixture of *Salmonella* (*S.* Typhimurium ATCC 14028 and *S.* Enteritidis ATCC 13076), the concentration of biofilm decreased to 44.28% and 58.14% when 7 log_10_ and 8 log_10_ PFU/mL phage cocktail was applied, respectively (Figure 6A). Phage cocktail achieved average 5.50 log_10_ and 6.42 log_10_ reduction of single *S.* Typhimurium (ATCC 14028) biofilm with respective titers of 7 log_10_ and 8 log_10_ PFU/mL on a stainless steel surface (Figure 6B). Similarly, treatment with the mixture of *Salmonella* (*S.* Typhimurium ATCC 14028 and *S.* Enteritidis ATCC 13076), resulted in decreasing biofilms concentration of at least 5.23 log_10_ CFU/mL and 5.77 log_10_ CFU/mL with phage cocktail titers 7 log_10_ and 8 log_10_ PFU/mL, respectively (Figure 6B).

## 4. Discussion

Phages are plentiful through surroundings, with approximately >10^31^ phages particles on the earth [70]. In this study, 42 phages were isolated from environmentally sourced water samples, which contained a low density of *Salmonella* strains [71,72]. It has been reported that sampling sites with low bacterial host density appear to have broad range lytic phage [73]. The selection of appropriate phages to be used in phage therapy based on spot test, and lytic activity against various *Salmonella* serovars [54,74]. Based on spot test results, phage LPSTLL, LPST94 and LPST153 showed broad lytic range activity as they could lyse 50–100% tested *Salmonella* serovars (*S.* Typhimurium, *S.* Enteritidis, *S.* Dublin, *S.* Kentucky, *S.* Paratyphi B etc.) including drug-resistant *Salmonella*. In the process of selecting the most effective phage, further screening of phages by lytic activity test was carried out [75,76]. Results of lytic activity indicated that LPSTLL, LPST94, and LPST153 had the strongest lytic capacity among all tested samples. The three phages LPSTLL, LPST94 and LPST153 tested in this study represented broad lytic range activity and high efficiency to inactivate the *Salmonella*.

From the lytic activity results, phage cocktail showed a higher lytic activity against *Salmonella* in vitro and could constantly inhibit the growth of *S.* Typhimurium ATCC 14028 and *S.* Enteritidis ATCC 13076 for up to 20 h compared with single applied phage LPSTLL, LPST94 and LPST153 that suppressed host growth for 12 h at MOIs of 0.1, 1, 10 and 100. Since different groups of phage recognize different receptors on host cell, a phage cocktail mix therefore could potentially delay the development of bacterial resistance or even prevent it. As the consequences of this, large proportion of bacteria will remain sensitive to certain phage cocktail candidates even after being infected, which will lead to a more severe viable count decline compared with singe phage treatment [77,78]. In contrast, single phage FGCSSa1, LPST10, LPST18, and LPST23 isolated by other researchers only revealed a 2–6 h inhibition with respect to host cell growth (*S.* Typhimurium PT160) at MOIs of 0.3–10 [54,71]. However, previously reported phage cocktail could inhibit their host growth for 12 h [79]. The above results are in accordance with other studies [45,75] that achieved higher inactivation by using phage cocktails, than that obtained with single-phage suspension. Mariann Landsberger and her coworkers investigated that one phage can cooperate to overcome CRISPR resistance of bacteria, leading to immunosuppression and leaving the host vulnerable to future, which allow other phage to infect and further lyse the bacteria [80]. Therefore, the phage cocktails approach has the potential to be one of the most promising choices to control *Salmonella.*

In this study, the three most virulent phages with the broadest host ranges within our collection were selected for establishment of phage cocktail for biocontrol applications and to reduce biofilm. As examined by TEM, three phages, LPSTLL, LPST94 and LPST153, belonged to the order of *Caudovirales* and family of *Siphoviridae*, *Ackermannviridae* and *Podoviridae,* respectively. Phages belong to these families all have the potential, as suggested by other literatures, to apply as biocontrol candidates against *Salmonella* and their biofilms [81,82,83,84,85,86].

In the phage stability study, it showed that phage cocktail effectivity remained stable over the tested periods both in milk and on chicken breast (Figure 3). However, small losses in phage titers in diverse food products (Chinese cabbage, chicken breast, mixed seafood and chocolate milk) were observed by other researches [52,87]. Our results suggested that phage cocktail is a suitable candidate for biological control of *Salmonella* in foods. Other researchers also showed that the phage cocktail could control pathogenic bacteria in foods, biofilms and food safety quality [88,89].

Application of the phage cocktail was predominantly effective against *Salmonella* in milk and on the surface of chicken breast, reducing the numbers of *Salmonella* counts down below the detectable limit (<1 CFU/100 μL) at both 4 °C and 25 °C with an MOI of 10000 and 1000. Because the high stability of phage cocktail could reduce the growth rate of bacteria at that temperature [90,91]. It has been demonstrated that, using high concentrations of phages generally achieved high reduction rates of pathogens [74,88,92]. When high titers are applied, phages are capable to absorb to the bacterial cells causing lysis to the cytoplasmic membrane without replication [10,93], reported as a process known as "lysis from without" [94]. Although very few study used a phage cocktail to control *Salmonella* in milk, some research applied a phage cocktail to reduce *E. coli* or *Listeria* and the bacterial counts dropped below the detectable limit in milk. In our study, phage cocktail application in milk led to the *Salmonella* count dropping below the detection limit when an MOI of 10000 and 1000 was applied, and this is consistent with the application of cocktails to reduce other bacteria [65,95]. Our results showed *Salmonella* counts (3 Log_10_ CFU/cm^2^) were eliminated completely with an MOI of 10000 and 1000 on chicken breast upon phage cocktail treatment. It was reported that, only 1 log CFU/g *Salmonella* viable count reduction was found either with single phage treatment at an MOI of 10000–1000000 [90,96] or with phage cocktail at an MOI of 10 [97] on chicken breast. The results demonstrated that the phage cocktail showed promise as biocontrol agents to control *Salmonella* in milk and on chicken breast compared with other similar studies and thereby could potentially reduce foodborne illness.

The biofilm study has shown that phage cocktail can infect *Salmonella* biofilm and has the potential to reduce tested *S.* Typhimurium and *S.* Enteritidis strains. The results suggested that phage cocktail treatment on two abiotic surfaces can effectively reduce the biofilms. It has been found that treatment with a phage cocktail eradicated post-treated biofilm in 96-well microplate (44–63%) and on stainless steel surface (ranging from 5.23 to 6.42 log_10_). Many types of research showed that effective biofilm eradications (ranging from 1 to 6 log), depend on the elements of the biofilm, the age of biofilm, phage effectiveness and length of treatment [98,99]. In this study, the data provided the proof of the principle that the application of phage cocktail could reduce the *S.* Typhimurium and *S.* Enteritidis in certain food types and to reduce biofilms on food contact surfaces that are important to maintain public health.

## 5. Conclusions

We isolated broad host lytic phages, LPSTLL, LPST94 and LPST153, prepared and characterized a phage cocktail with a broad spectrum of activity against diverse *Salmonella* serovars. Our results revealed that treatment of artificially contaminated foods with a phage cocktail completely lysed and eliminated the contaminating *Salmonella*, as well as eradiated the biofilms on food contact surfaces, indicating that this phage cocktail is a prime candidate for the biological control of *Salmonella* and can inactivate the biofilms that are resistant to traditional approaches.

## Figures and Tables

**Table 1:** List of bacterial strains used in this study.

Bacterial Strains	Strain ID Number	Numbers of Strains	Source of Strains
*S.enterica* serovar Typhimurium	ATCC 14028, ATCC 13311	2	ATCC
	UK-1, ST8, SGSC 4903, SL 1344, LT2	5	LS
*S.enterica* serovar Enteritidis	ATCC 13076	1	ATCC
	SJTUF 10978, SJTUF 10984	2	SJTU
	LK5-3820, SGSC 4901	2	LS
*S. enterica* Serovar Pullorum	CVCC 519	1	LS
*S.enterica* serovar Dublin	3710,3723	2	LS
*S. enterica* subsp. *enterica* serovar Anatum	ATCC 9270	1	ATCC
*S. enterica* subsp. *arizonae*	CDC 346-86	1	CDC
*S. enterica* subsp. *enterica* serovar Javiana	CVM 35943	1	LS
*S. enterica* subsp. *enterica* serovar Kentucky	CVM 29188	1	LS
*S. enterica* serovar Newport	E20002725	1	CDC
*S. enterica* serovar Paratyphi B	CMCC 50094	1	CMCC
*S. enterica* serovar Choleraesuls	ATCC 10708	1	ATCC
Drug resistant *S. enterica* serovar Typhimurium	LST10, LST11, LST12, LST13, LST14, LST15, LST16, LST17, LST18, LST19	10	LS
Drug resistant *S.* *enterica* serovar Enteritidis	LSE6, LSE7, LSE8, LSE9, LSE10, LSE11, LSE12, LSE13, LSE15	9	LS
*E. coli*	BL21, DH5α	2	TB
	ATCC 933	1	ATCC
	F18AC, C83715, T10	3	LS
*A. hydrophila*	ZYAH72, ZYAH75, J1, D4	4	LS
*S. aureus*	ATCC 6538, ATCC 8095, ATCC 29213	3	ATCC
*Listeria*	ATCC 1914	1	ATCC

Abbreviation: ATCC, American Type Culture Collection; LS, Lab Stock; SJTU, Shanghai Jiao Tong University; CDC, Centers for Disease Control and Prevention; TB, TransGen Biotech; CMCC, National Center for Medical Culture Collection.

**Table 2:** Sensitivity of different *Salmonella* serovars and other bacterial strains to lyse by selected phages determined by spot testing.

Bacterial Strains	% of Positive Spot Test against *Salmonella* Serovars and Other Bacterial Strains						
	LPST81	LPSTLL	LPST89	LPST94	LPST109	LPST115	LPST153
*Salmonella* serovars							
Typhimurium (*N* = 7)	71.4	100	100	100	100	71.4	85.7
Enteritidis (*N* = 5)	60	100	40	100	60	20	80
Dublin (*N* = 2)	0	100	0	100	50	0	50
Choleraesuls (*N* = 1)	0	100	0	100	0	0	100
Newport (*N* = 1)	0	100	100	100	0	0	0
Paratyphi B (*N* = 1)	100	100	0	100	100	0	100
Anatum (*N* = 1)	0	100	0	100	0	0	0
Pullorum (*N* = 1)	0	100	0	100	0	0	100
Javiana (*N* = 1)	0	100	100	100	0	0	100
Kentucky (*N* = 1)	0	100	0	100	0	0	0
*S. arizonae* (*N* = 1)	0	100	0	100	0	0	100
Drug resistant *Salmonella* serovars							
Typhimurium (*N* = 10)	60	100	90	100	70	10	90
Enteritidis (*N* = 9)	11.1	100	33.3	100	22.2	11.1	88.9
Other bacterial strains							
*E. coli* (*N* = 6)	0	0	0	0	0	0	0
*A. hydrophila* (*N* = 4)	0	0	0	0	0	0	0
*S. aureus* (*N* = 3) *Listeria* (*N* = 1)	0 0	0 0	0 0	0 0	0 0	0 0	0 0

**Table 3:** Efficiency of plating (EOP) by phages (LPSTLL, LPST94, LPST153 and cocktail) against different *Salmonella* serovars.

Bacterial Strains	LPSTLL	LPST94	LPST153	Cocktail	Strains	LPSTLL	LPST94	LPST153	Cocktail
*S.enterica* serovar Typhimurium					Drug resistance *S.* *enterica* serovar Typhimurium				
ATCC 14028	1	1	0.18	1	LST10	0	0.1	0.004	0.26
ATCC 13311	1	1	Host	1	LST11	0.19	0.4	0.003	0.48
UK-1	Host	Host	0.17	1	LST12	0.1	0.3	0.007	66
ST8	1	1	0.1	1	LST13	0.02	0.45	0.009	0.89
SGSC 4903	1	1	1	1	LST14	0.1	0.1	0.006	38
SL 1344	1	1	1	1	LST15	0.006	0.15	0.1	0.32
LT2	1	1	1	1	LST16	0.007	0.1	0.002	0.22
*S.enterica* serovar Enteritidis					LST17	0	0.4	0.005	0.73
ATCC 13076	0.1	0.4	0.1	0.6	LST18	0.009	0.1	0.010	0.24
SJTUF 10978	0.02	0.24	0.19	0.4	LST19	0	0.2	0.016	0.31
SJTUF 10984	0.1	0.19	0.17	0.3	Drug resistance *S. enterica* serovar Enteritidis				
LK5-3820	0.005	0.4	0.003	0.35	LSE6	0	0.005	0	0.23
SGSC 4901	0.18	0.3	0.12	0.5	LSE7	0.017	0.1	0.004	0.37
*S.enterica* serovar Dublin					LSE8	0.003	0.3	0.1	0.55
3710	0	0.19	0	0.3	LSE9	0.015	0.4	0	0.62
3723	0	0.2	0	0.25	LSE10	0	0.1	0	0.23
*S. enterica* serovar Choleraesuls					LSE11	0.1	0.006	0	0.29
ATCC 10708	0.009	0.16	0	0.3	LSE12	0	0.2	0	0.27
*S. enterica* serovar Newport					LSE13	0	0.007	0	0.24
E20002725	0	0.15	0	0.23	LSE15	0.14	0.1	0.013	0.51
*S. enterica* serovar Paratyphi B									
CMCC 50094	0.900	0.2	0.19	0.44					
*S. enterica* Serovar Pullorum									
CVCC 519	0.017	0.2	0.1	0.67					
*S. enterica* subsp. *Enterica* serovar Javiana									
CVM 35943	0.012	0.1	0.003	0.24					
*S. enterica* subsp. *enterica* serovar Anatum									
ATCC 9270	0	0.007	0	0.21					
*S. enterica* subsp. *enterica* serovar Kentucky									
CVM 29188	0.4	0.2	0.1	0.76					
*S. enterica* subsp. *arizonae*									
CDC 346-86	0.004	0.2	0	0.27					

EOP 0.5 to 1.0, high efficiency; EOP 0.2 to <0.5, moderate efficiency; 0.001 to <0.2, low efficiency; and <0.001, inefficient.
